# Disseminated Histoplasmosis: A Rare Cause of Pancytopenia in an Immunocompromised Patient

**DOI:** 10.7759/cureus.25966

**Published:** 2022-06-15

**Authors:** Kalyani Avva, Brandon Wu, Leslie Cler

**Affiliations:** 1 Internal Medicine, Methodist Health System, Dallas, USA

**Keywords:** opportunistic infection of oncology patient, histoplasmosis, opportunistic infection, histoplasma capsulatum, disseminated histoplasmosis

## Abstract

*Histoplasma capsulatum *is a dimorphic fungus endemic to North and South America. This organism's ubiquity outside the traditionally defined region of the Mississippi and Ohio River Valley makes it an important yet often forgotten cause of systemic inflammatory disease. Progressive disseminated histoplasmosis is an uncommon opportunistic infection, largely affecting immunocompromised individuals with defects in T-cell immunity. The initial manifestations of disseminated histoplasmosis present non-specifically with symptoms such as fever, malaise, anorexia, and weight loss. Given this fungi's endemic nature, disseminated histoplasmosis is an essential disease for physicians to diagnose accurately. Diagnosis can be established through antigen detection in the blood or urine, although the gold standard is tissue diagnosis or fungal culture. Treatment of mild to moderate disease consists of an itraconazole regimen for a year, yet severe disease requires an additional 14-day induction therapy with amphotericin B. We present a case of disseminated histoplasmosis in a breast cancer patient, recently treated with neoadjuvant chemotherapy, who presented with new-onset pancytopenia.

## Introduction

*Histoplasma capsulatum (*H. capsulatum) is a dimorphic fungus endemic to North and South America, particularly the Mississippi and Ohio River Valleys. H. capsulatum is linked to the fungal respiratory infection histoplasmosis, which typically portends a self-limiting course in previously healthy individuals. Progressive disseminated histoplasmosis is an uncommon opportunistic infection. Affected individuals are typically immunocompromised with defects in T-cell immunity. The initial manifestations are relatively non-specific (eg., fever, malaise, anorexia, and weight loss), making it a challenging disease to diagnose. We present a case of disseminated histoplasmosis in a breast cancer patient recently treated with neoadjuvant chemotherapy who presented with new-onset pancytopenia [1].

This article was previously presented as a poster at the Annual Meeting of the Texas Chapter of the American College of Physicians in November 2021.

## Case presentation

**Figure 1:**
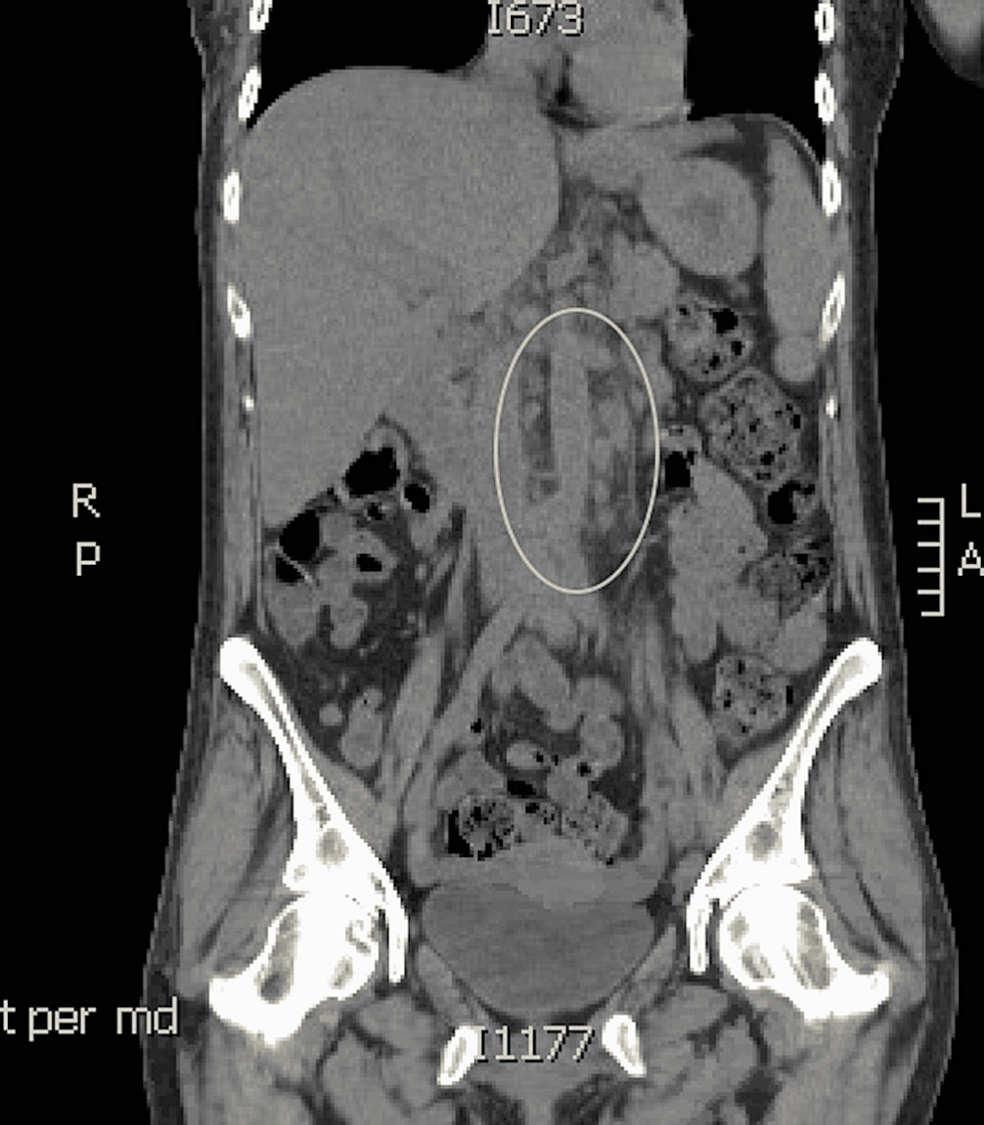
A computed tomography scan of the abdomen and pelvis. The peri-aortic and retroperitoneal lymph nodes are circled.

**Figure 2:**
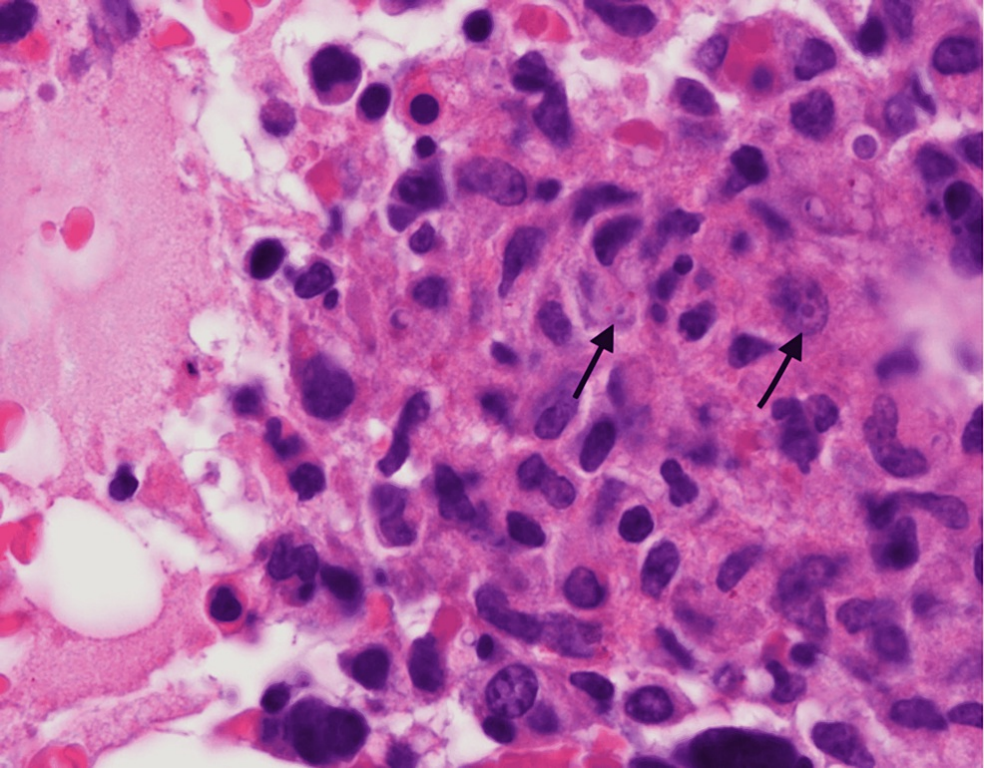
Bone marrow biopsy (H&E stain) showing Histoplasma infected macrophages

**Figure 3:**
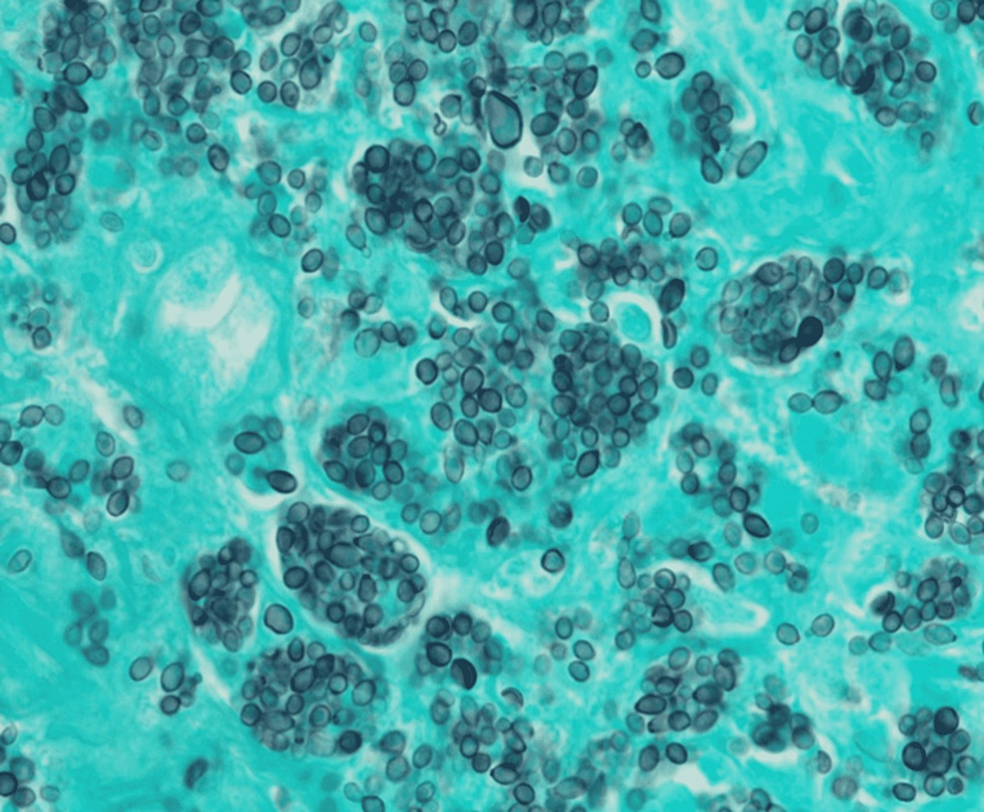
Core lymph node biopsy showing Histoplasma infiltration (Grocott methenamine silver stain).

The patient was a 47-year-old female with a past medical history of Stage IIb right-sided triple-negative breast cancer. She was status post neoadjuvant chemotherapy with Adriamycin and cyclophosphamide. One month before hospital presentation, she underwent a mastectomy with axillary lymph node dissection and underwent subsequent adjuvant chemotherapy with paclitaxel treatment. She presented to the hospital with 48 hours of intractable nausea and vomiting and a two-week history of fatigue and Physical malaise exam at the time of admission was unremarkable, but 24 hours after admission, she developed a fever of 39°C and her labs showed a white blood cell count of 2,500 cells/µL, a hemoglobin level of 7.4 g/dL, and a platelet count of 64,000 platelets/µL of blood. Computed tomography imaging of her chest, abdomen, and pelvis showed numerous enlarged periaortic and retroperitoneal lymph nodes (Figure 1). The patient subsequently underwent bone marrow (Figure 2) and lymph node (Figure 3). Biopsy showed a high disease burden with Histoplasma, and a positive Histoplasma urine antigen test further supported her diagnosis. She was started on induction therapy with a 14-day course of amphotericin B and then transitioned to a year-long course of itraconazole.

## Discussion

Although Histoplasma is prevalent in North and South America, with endemic proportions seen in the Mississippi and Ohio River Valleys of the United States, reports show that this fungus is also prevalent in regions of India, China, and Southeast Asia, and Sub-Saharan Africa [2]. Regrettably, outside of non-hyperendemic regions, histoplasmosis is under-recognized by clinicians and often confounded with other disease processes. Given the increasing rates of international travel, this is a significant disease for clinicians to diagnose accurately. This is especially important given the high transmissibility of H. capsulatum during outdoor labor and recreation [3].

Transmission of H. capsulatum to a human host occurs through spore inhalation and subsequent macrophage phagocytosis. Infection arises when this pathogen can evade degradation in the macrophages by deactivating reactive oxygen and nitrogen species and preventing lysosomal acidification. Additionally, macrophage inactivation enables H. capsulatum to lie dormant in the host for many years, thus promoting latent disease [4]. In rare cases, when Histoplasma infection triggers macrophage apoptosis, increased levels of IL-10 and TNF-α are generated. This induces granulomatous inflammation and dissemination to other organs [5].

Fortunately, approximately 90% of exposed individuals remain either asymptomatic or have self-limiting symptoms. Those exposed to Histoplasma may develop active disease via three mechanisms: acute infection, reactivation, or re-infection. For most immunocompetent individuals, the disease course is relatively mild, consisting of an acute flu-like pulmonary illness. In contrast, those with defects in T-cell immunity, such as individuals with HIV or on certain immunosuppressive medications, are at high risk for disseminated histoplasmosis given the unchecked granulomatous inflammation. In its early stages, disseminated histoplasmosis has non-specific main symptoms, including fever, weight loss, and fatigue. Laboratory findings may show pancytopenia, elevated liver function, and elevated lactate dehydrogenase levels. Initial chest imaging can even be negative. If disseminated histoplasmosis is not detected expediently, disease progression can manifest as Histoplasma-associated colitis, Histoplasma meningitis, or papular cutaneous eruptions [5]. The prompt diagnosis and treatment of disseminated histoplasmosis are critical since delays in treatment are associated with a high mortality rate, and untreated disease is uniformly fatal [3]. Diagnosis can be established through antigen detection in the blood or urine, although the gold standard is tissue diagnosis or fungal culture [1]. Treatment for mild to moderate disseminated histoplasmosis is with itraconazole for at least 12 months. For severe disease, as seen in this patient, treatment consists of amphotericin B induction therapy for two weeks, followed by a 12-month course of itraconazole [6].

Our case is unique because the patient had recently completed neoadjuvant chemotherapy with Adriamycin and cyclophosphamide, followed by surgical treatment and subsequent paclitaxel treatment. These drugs are inherently cytotoxic but not associated with frank helper T-cell suppression, unlike TNF-α inhibitors which are more commonly associated with reactivation of latent Histoplasma [1,5]. This case was also notable because the patient initially presented with fever and pancytopenia in the absence of recent travel or prior residence in a hyper-endemic area. The patient's oncologic and travel history initially placed drug-related marrow suppression or metastatic disease at the top of the differential. Additionally, the patient presented with a constellation of intractable nausea, vomiting, and hypotension, which is very unusual for disseminated histoplasmosis but is common in oncology patients. These factors can make diagnosing disseminated histoplasmosis in the immunocompromised population difficult because other infectious agents are likely to be suspected first [7]. Nevertheless, clinicians need to consider disseminated histoplasmosis on the differential when treating immunocompromised patients in non-endemic areas.

## Conclusions

The lower prevalence of H. capsulatum outside of the traditionally defined region of the Mississippi and Ohio River Valleys makes it an important, yet rarely considered, cause of systemic inflammatory disease. Given its relatively non-specific initial presentation, a diagnosis of disseminated histoplasmosis is often overlooked in non-endemic areas. This case highlights the significance of a timely diagnosis of disseminated histoplasmosis in an immunocompromised host who resided outside of a hyper-endemic area. Given the almost worldwide distribution of H. capsulatum and the increasing frequency of patients with immunosuppression, this is a significant disease for clinicians to diagnose.
